# Non-invasive measuring of biopotentials of the ciliary muscle during accommodation in emmetropes

**DOI:** 10.1038/s41598-025-04165-3

**Published:** 2025-06-03

**Authors:** Sven Schumayer, Bishesh Sigdel, Mohamed Ali Jarboui, Eberhart Zrenner, Volker Bucher, Torsten Straßer, Sandra Wagner

**Affiliations:** 1 Faculty Mechanical and Medical Engineering (MME), Institute for Microsystems Technology (iMST), Furtwangen University, Furtwangen, Germany; 2 Institute for Ophthalmic Research, University of Tuebingen, Tuebingen, Germany; 3 Core Facility for Medical Bioanalytics, Institute for Ophthalmic Research, University of Tuebingen, Tuebingen, Germany; 4 University Eye Hospital Tuebingen, Tuebingen, Germany

**Keywords:** Contact lens electrode, Biopotential, Ciliary muscle, Accommodation, Electrophysiology, Preclinical research, Proteomics

## Abstract

To see near objects clearly, the ciliary muscle shapes the human eye’s crystalline lens to adjust its refractive power, a process known as accommodation. This contraction of the ciliary muscle also results in an electrical potential change. Previous work from the 1950s and 1960s reported electrical voltages in the microvolt range that were attributed to the accommodating ciliary muscle, however without clarifying the interaction between lens and muscle. Here, we present data of 12 emmetropic participants using a custom-developed scleral contact lens electrode which enables to record accommodation-dependent biopotentials of the ciliary muscle with an accuracy up to the millivolt range. Therefore, participants alternately shifted their focus from far to various near targets while the biopotentials of the ciliary muscle and the actual refractive change of the crystalline lens were recorded by a contact lens electrode and an eccentric infrared Photorefractor. In addition, the impact of confounding biopotentials such as squinting and eye movements was investigated. Our research points to a potentially new objective method of measuring accommodative change. Understanding these biopotentials could lead to the development of self-focusing visual aids as an alternative way of vision correction in presbyopes.

**Supplementary Information:**

The online version contains supplementary material available at 10.1038/s41598-025-04165-3.

## Introduction

To shift the focal point from a far to a near target, the ciliary muscle in the human eye increases the dioptric power of the crystalline lens, a process known as accommodation^1^. Today’s understanding of accommodation is based on Helmholtz’s theory of 1855^2^ with Fincham’s amendments^3^ and remains a topic of ongoing debate^4^. The dominant stimuli triggering the change of the refractive power are blur and vergence^5–7 ^resulting in what is commonly referred to as a “near triad” of accommodation, convergence and pupil constriction^6,7^. Helmholtz postulated that the contraction of the triangular ciliary muscle, which is concentrically arranged around the lens, and in particular its differently arranged muscle fibers, causes the lens to become more spherical and translate the anterior lens surface forward^8,9^. This is obtained by zonular fibers, a suspensory ligament, that reduces the tension between the ciliary body and the elastic lens capsule^8,10^. However, the ability to accommodate diminishes with age, typically becoming noticeable between the age of 40 to 45 years^11^. This loss of function, known as presbyopia, is assumed to be due to physiological changes of the crystalline lens in terms of geometry and material properties, which are still the subject of current research^12,13^. Several vision correction methods are currently available, including spectacles^14,15 ^contact lenses^15 ^ophthalmic surgeries^14,16 ^and pharmaceutical treatments^14,17,18^. Each of these approaches has its own advantages and limitations, with some still requiring further research^19^. All of these options have in common that they only treat the symptoms of presbyopia and do not restore natural accommodation. In particular, they are unable to close the interrupted feedback loop of the accommodative system. This is due to the age-related non-functioning crystalline lens^20 ^resulting in an inability to close the feedback loop in presbyopes even with the intact ciliary muscle^21^. The ability to measure these signals from the human ciliary muscle using, for example, contact lens electrodes, was demonstrated by various researchers nearly a century ago^22–26^. Schubert et al., 1955 were the first to record bioelectric signals (0.3 mV) in eight emmetropes and one presbyope, which they attributed to the ciliary muscle using exclusion proceedings^23^. In 1958, Alpern recorded voltage changes of up to 0.2 mV during a focus change from far to near, which were positively correlated with the accommodation effort. Alpern excluded electrical muscle artifacts based on the experimental setup and demonstrated that the contributions of the pupil response were negligible^26^. Hagiwara and Ishikawa (1962) measured potential changes of up to 0.15 mV in the accommodated state using a contact lens electrode, with these changes decreasing during cycloplegia^25^.

The aim of the present study was to investigate the electrical potentials of the ciliary muscle during the controlled presentation of accommodative stimuli at various distances, using a previously described novel bipolar scleral contact lens electrode^27^. Biopotentials were recorded in a group of young healthy emmetropes while simultaneously measuring changes in the refractive state of the crystalline lens. Potential confounding sources of the recorded biopotentials other than the ciliary muscle were analyzed by eliciting pupillary responses, gaze changes, eye squinting, and by paralyzing the ciliary muscle using cycloplegia. Additionally, a proteomic analysis was performed to investigate if the contact lens wear triggers any major inflammatory response. The ability to reliably record the electrical potentials provides a new tool for advancing the research on the ciliary muscle, extending beyond just presbyopia. Ultimately, these biopotentials could be used to close the interrupted feedback loop of the accommodative system in presbyopia^20^ by controlling an artificial lens to restore dynamic accommodation.

## Results

The refractive error in the spherical equivalent was − 0.06 ± 0.33 D (mean ± s.d.) and the amplitude of accommodation in the left eye was 8.2 ± 1.39 D. All participants presented with monocular visual acuity of 20/20 or better. The right eye was the dominant in 8 out of 12 participants.

### Biopotentials for different viewing distances

**Fig. 1:**
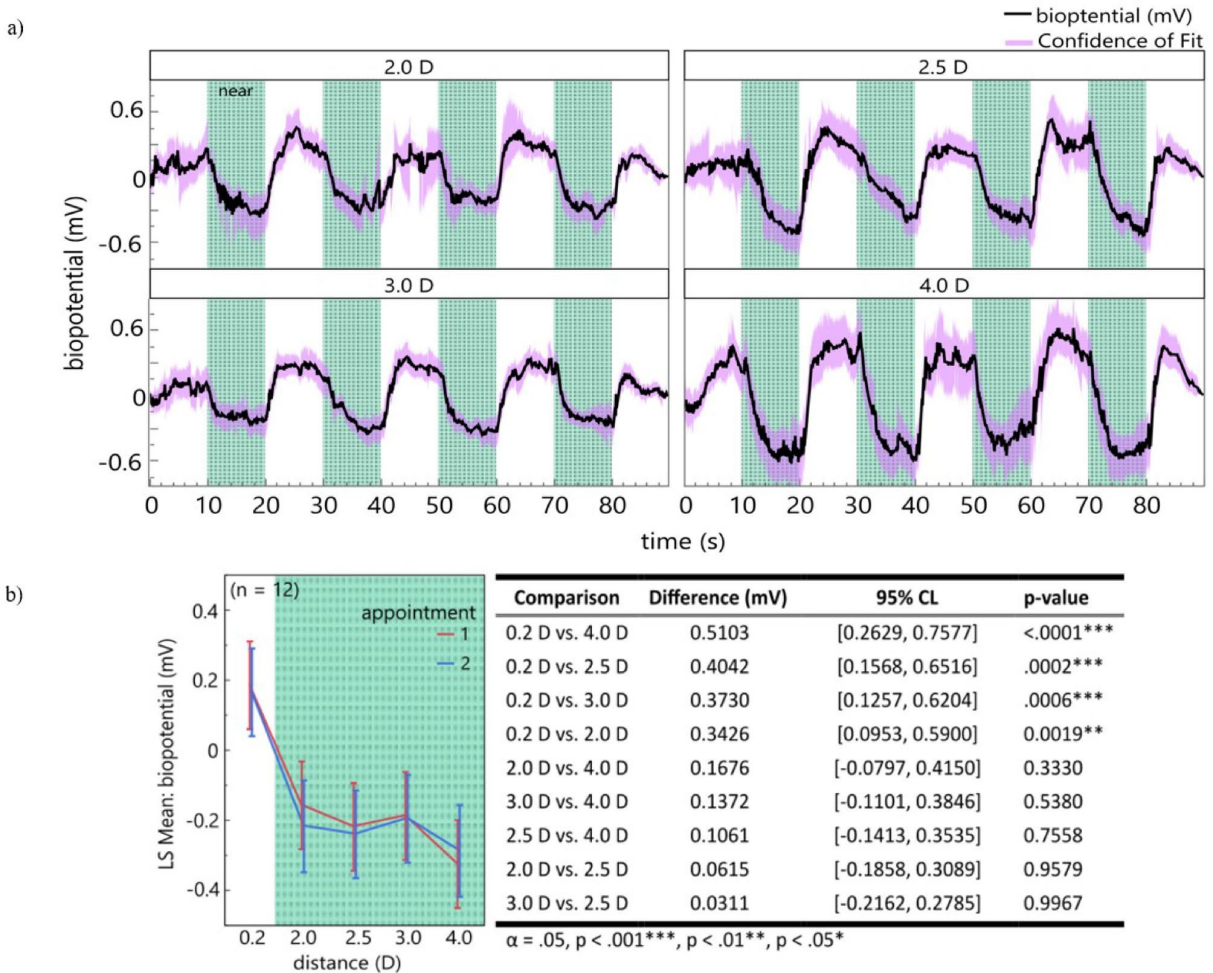
Biopotentials for different viewing distances: (**a**) The biopotential was filtered (bandpass, 2nd order, 0.025–0.575 Hz) and arranged between 0 to 1. Moving average of the biopotentials (width:10) of the two measurement appointments for all participants^x^ with the purple shaded area as the 95%-confidence of fit. Green bars represent the 10 s period of near vision at the different distances (2.0, 2.5, 3.0, 4.0 D). (**b**) (left) The LS mean plot of the electrical potential amplitude over the different accommodative demands for the first and second appointment. (Right) Results of the post hoc Tukey-HSD test, comparing the mean amplitude between different accommodative demands. ^x^Measurements (ID:02 2.0D & ID:02 4.0D) were excluded.

An overview of the measured biopotentials at the different distances is shown in Fig. 1a. The LMM (R²= 0.4) reveals a highly significant influence of the fixed effect accommodation demand (F(4, 97.01) = 9,3966, *p* < 0.0001) while the fixed effect *session* (F(1, 97.67) = 0.1247, *p* = 0.7248) and the resulting interaction effect (F(4, 97.26) = 0.148, *p* = 0.9635) between *accommodation demand* and *session* showed no significant influence. The estimated least-squares means show that the accommodation demand of 4.0 D led to the highest biopotentials (-0.325 mV, 95% CI: [-0.45, -0.199]), while 2.0 D (-0.157 mV, 95% CI: [-0.282, -0.032]), 2.5 D (-0.219 mV, 95% CI: [-0.344, -0.093]) and 3.0 D (-0.187 mV, 95% CI: [-0.313, -0.062]) showed values that were similar in range. Compared to the far target at a distance of 0.2 D (0.186 mV, 95% CI: [0.06, 0.311]), a post hoc Tukey-HSD test shows a high significance (cf. Figure 1b) between the far and every near target. Similar results are seen in the LMM (R²= 0.86) of the measured refractive changes of the participants (cf. Supplementary Figure S1). This model indicates a highly significant influence of the fixed effect *accommodation demand* (F(4, 97.01) = 10.3704, *p* < 0.0001), while neither *session* (F(1, 97.03) = 0.1520, *p* = 0.6975), nor their interaction (F(4, 97.01) = 1.0234, *p* = 0.3992) showed any significant influence. However, discrepancy was seen by the random effect *subject.* While 82.57% of the total variance of the mean refraction change could be explained by the random effect subject, it was 0.887% for the mean biopotential. A comparison between the normalized mean biopotential values and the change in refraction shows a weak positive correlation within the first session (*r* = 0.208; *p* = 0.1108).

**Fig. 2:**
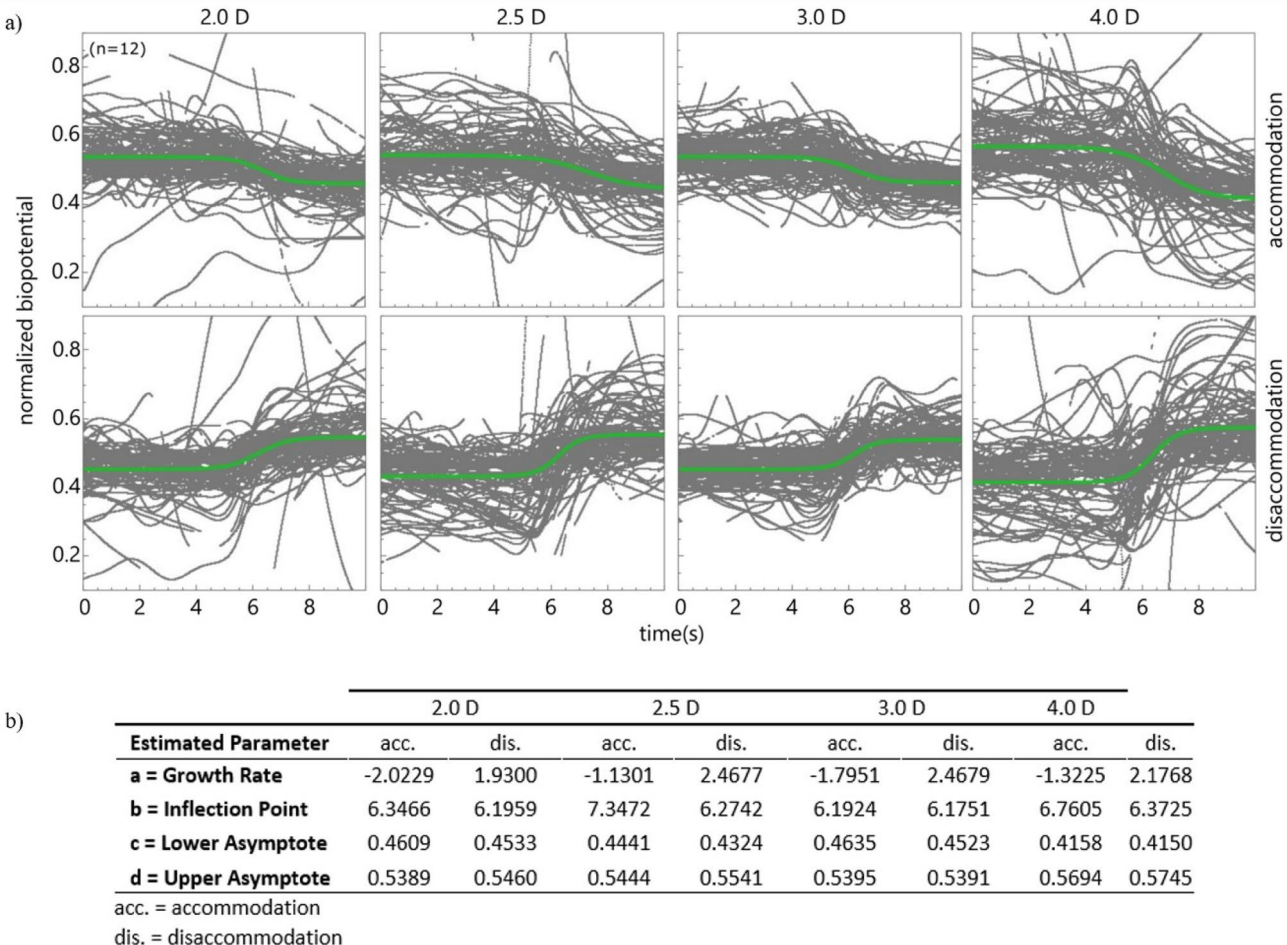
Curve fitting: (**a**) Logistic sigmoid curve fitting of normalized biopotential responses during accommodation (top row) and disaccommodation (bottom row) at different stimuli (2.0, 2.5, 3.0,  4.0 D). Each plot presents the individual responses to each sequence (grey lines, *n* = 12) with the fitted sigmoid curve (solid green line) over time (seconds). The time interval covers 5 s before and after the respective stimulus. (**b**) The table contains the estimated parameters from the sigmoid fitting showing the dynamic properties of the biopotential responses, with notable differences in rate of change (b.a) between dis- and accommodation, whereas lower (b.c) and upper (b.d) asymptote, stays mostly similar. The inflection point (b.b) seems to be slightly faster during disaccommodation.

Modeling the normalized biopotentials with logistic sigmoid functions revealed differences in the dynamic properties between accommodation and disaccommodation (Fig. 2): While both are not correlated with accommodative effort (2.0, 2.5, 3.0, 4.0 D), disaccommodation shows a higher growth rate compared to accommodation, indicating a faster response when focusing from near to far. Accordingly, the time of maximum change is slightly shorter for the disaccommodation, while the maximum and minimum response remain constant for all far-near and near-far responses.

### Measurement of confounding biopotentials

**Fig. 3:**
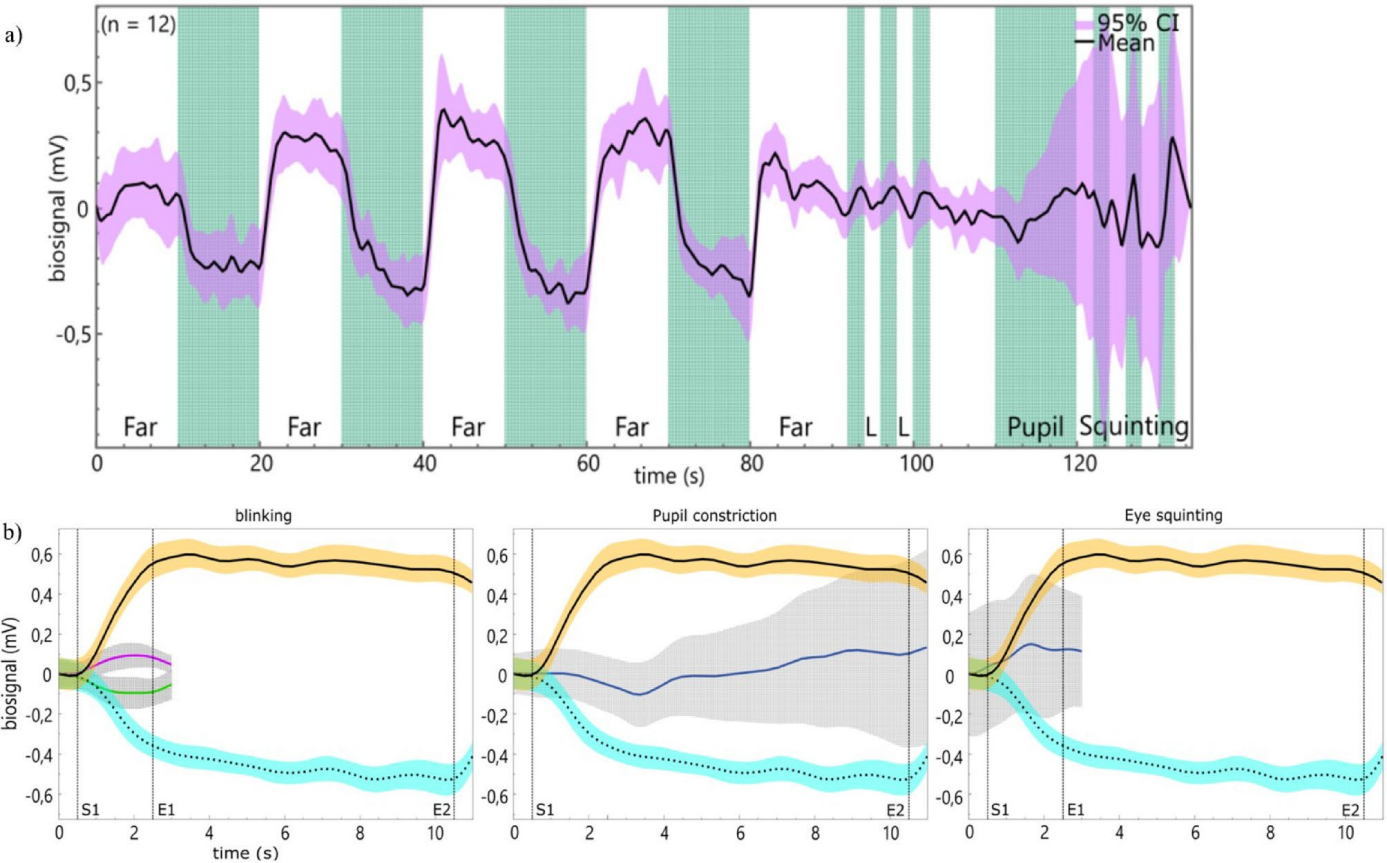
Confounding biopotentials: (**a**) An overview of the entire confounding signal measurement, in which the 2° horizontal eye deflection to the right and left (L), the forced pupil constriction, and the eye squinting were measured after an iterative change between far and near. The y-axis represents the biopotentials in millivolt and the black line the mean for the two measurement appointments for all participants, while the 95% confidence interval is shown as the purple shaded area. Individual sequences are marked in green. The plots in (**b**) show different confounding signal means compared to the accommodation-related biopotentials together with the 95% confidence interval. Accommodation is shown by the black dotted line, while disaccommodation is represented by the black line and orange confidence intervals. The start (S1 at 0.5 s) and endings of the sequence (E1 at 2.5 s; E2 at 10.5 s) are marked by the vertical dashed lines. The signal progression during horizontal eye movement to the left is shown as a green (-) and to the right as a purple (+) line.

The course of the biopotential during the entire measurement of experimentally-induced confounding factors shows a signal characteristic which is distinguishable from the accommodation-related signals, noticeable in signal shape, amplitude level, and rate of change. Figure 3a shows the negative response at a rate of -167.6 µV/s reaching an average of -0.211 ± 0.313 mV after changing accommodation from far (0.2 D) to near (3.0 D), and a positive response at 271.4 µV/s reaching 0.162 ± 0.372 mV after disaccommodation. In contrast, the rate of change of the signals resulting from horizontal gaze change (left to right: 37.5 µV/s; right to left: -36.3 µV/s), pupil constriction (-27 µV/s), and squinting (41 µV/s) is much lower. The entire measurement of the horizontal gaze change combined with the biopotential can be found in the Supplementary Figure S2. Furthermore, the signals exhibit greater variability between repetitions compared to those resulting from accommodation or disaccommodation, as indicated by the 95% confidence intervals (Fig. 3b).

### Cycloplegia

**Fig. 4:**
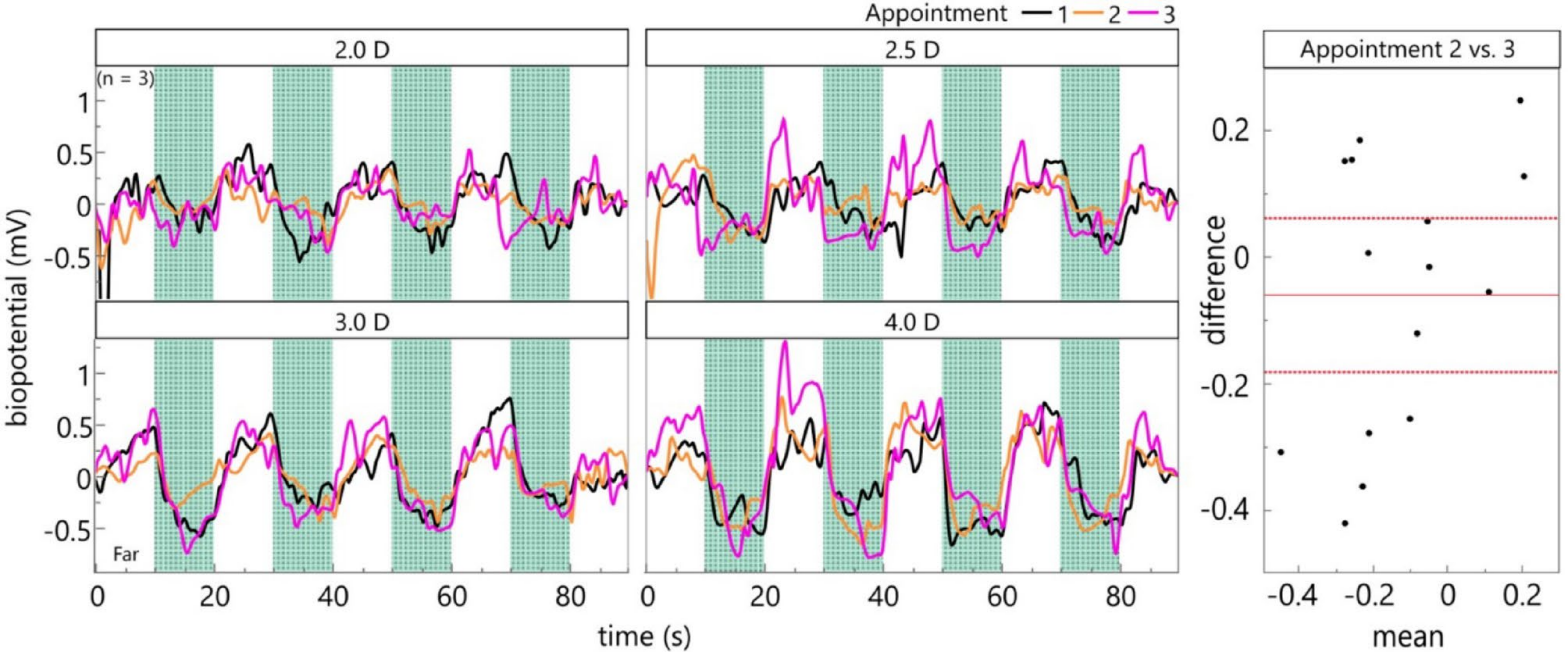
Cycloplegia: Left: Averaged biopotentials of three participants for each appointment and accommodation demand. Right: Bland-Altman plot for the biopotentials shows no significant difference between the second and the third appointment (under cycloplegia) for the three participants.

During cycloplegia, all 5 volunteers were unable to accommodate with their treated right eye, shown by an amplitude of accommodation far below 2 diopters. Contrary to expectations, however, the biosignals were only extinguished in 2 of the 5 participants (Fig. 4 left). A Bland-Altman analysis reveals no statistically significant differences between the mean amplitudes of the second and those of the third appointment under cycloplegia (t(14) = -1.051, *p* = 0.3112, two-tailed, cf. Figure 4 right). The mean biopotential velocity for the three volunteers, during disaccommodation from 4.0 D to 0.2 D, is 283.5 µV/s and for accommodation − 315 µV/s after cyclopentolate application. Refractive change in the untreated left eye is unaffected and comparable to results before cycloplegia, also showing no significant difference between the second and third appointment (t(14)= -0.176, *p* = 0.8629, two-tailed).

### Proteomics analysis

**Fig. 5:**
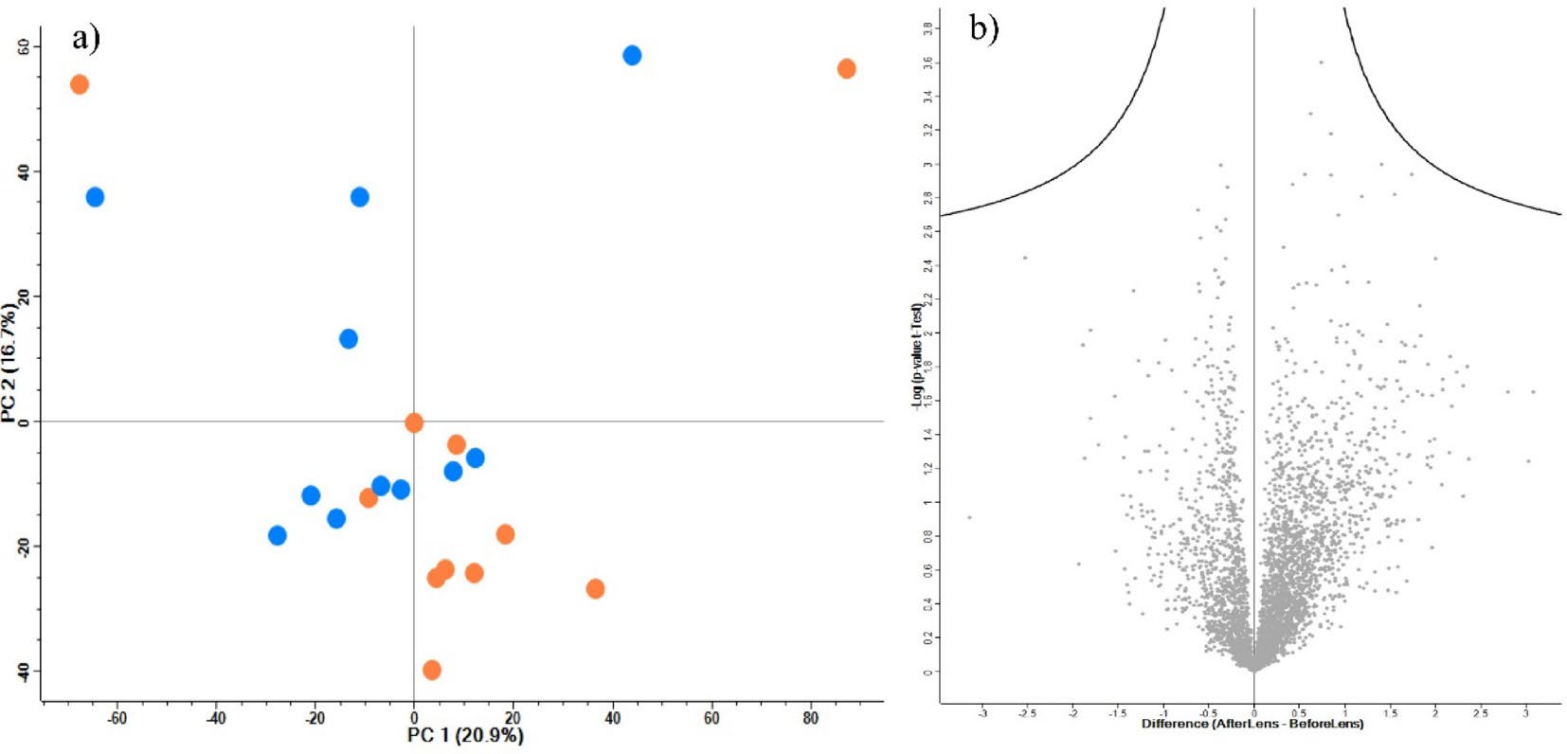
Proteomics analysis: (**a**) The PCA plot shows no clear segregation between the proteome before (blue dots) or after (orange dots) contact lens electrode wear. (**b**) Volcano plot of differential proteins abundance analysis using two samples t-test shows no significant changes (two-sided student t-test using permutation-based truncation FDR < 0.05).

Using DIA proteomics analysis, a total of 3738 proteins from 22 samples were identified and quantified. Principal component analysis (PCA) showed no clear clustering between the samples before or after lens wear. Furthermore, a paired two-sample t-test did not identify any proteins differentially changing in abundance between the 2 groups (before and after). These results suggest that the contact lens electrode wear did not trigger any major response that could lead to an increased abundance of specific proteins as a response mechanism to the wear of the scleral contact lens electrode (Fig. 5).

## Discussion

Using a newly developed bipolar scleral contact lens electrode^27 ^we were able to record electrical potentials from the ciliary muscle during accommodation in 12 emmetropic volunteers, which are consistent with results from previous literature^22–26,28^. In contrast to those, we measured the signal during an altering far-near-far stimulus together with the actual refractive change, using an eccentric infrared photorefraction. We did find a significant difference between the far and every near stimulus (*p* < 0.0001), however, the amplitude between different near targets did not differ significantly. This signal progression was already described in previous work^26^ and this kind of behavior was seen for the ciliary muscle thickness changes during accommodation^29^. However, we believe that the amplitude of the biosignal may not be the key parameter for detecting finer accommodative changes. We hypothesize that the accommodative apparatus (including the crystalline lens, the ciliary muscle, and the retina as a blur detector), is a closed-loop system in which the ciliary muscle continuously adjusts the curvature of the lens as long as blur is present on the retina. Once the blur is minimized, the muscle will likely stop contracting and consequently, the biopotential change will stop. For a biomimetic visual aid with a tunable lens, the presence of this biopotential change is of significance.

Interestingly, the sigmoidal fitting indicates that the rate of change in biopotentials from near to far was higher compared to far to near, for near vision target greater than 2.0 D, which is consistent with findings reported in the literature for changes in refractive power^30,31^. The determined inflection point - the time of the strongest change - is slightly earlier in disaccommodation compared to accommodation. A non-significant (*p* = 0.8064) difference was seen between the two measurement appointments, revealing the repeatability of the biopotential measurement. The inter variability, known from measurements of the refractive changes, could also be observed for the biopotentials. One possible explanation is that differences in crystalline lens curvatures of individuals^9^ result in different accommodative efforts. It is also known that the lag of accommodation varies among individuals^30^ as does the tonic accommodation^32,33^ and the individuum’s ability to sympathetically control the accommodation^34^. In terms of electrophysiology, the fit of the lens on the eye surface of the participants is also an essential factor, as this defines the electrical contact area. Studies^35,36^ have already established a concentric asymmetry and inter variability of the sclera. The polarity of the biopotential, becoming more negative during accommodation, can be explained by the connection of the electrode to the detection unit. While Schubert et al. (1955) and Hagiwara Ishikawa (1962), described an increasing positive polarity with a far-to-near stimulus, Alpern et al. (1958) described the opposite. Nonetheless, all three described an accommodation-related voltage change between 0.15 and 0.3 mV. This is consistent with this study, in which the 4.0 D accommodation demand resulted in the highest amplitudes of -0.32 ± 0.629 mV (mean ± s.d.). However, in some participants (e.g., ID: 08), amplitudes as high as -1.9 mV were measured.

Presenting the participants different visual stimuli, we could show that those biopotentials caused by eye movements, squinting or pupil constriction differ noticeably in signal characteristics compared to accommodation driven ones, which was also mentioned, however not shown, in the work of Hagiwara and Ishikawa^25^. The calculated rate of change shows very similar behavior to the observations made when measuring different near distances. Interestingly, the horizontal eye movement between right and left differed in polarity and showed a waveform already described by Jacobson et al. (1958). Considering this shape, amplitude height, and the calibration of the setup before the measurement to minimize possible eye movements, the extraocular muscles can be neglected as the origin of the accommodation-related biosignals. Eye squinting caused the contact lens electrode to move, leading to a shift in the ion concentration and thus to a change in polarity. Taking the short ion-change into account, a spiking signal is expected. Additionally, the biosignals recorded during pupil constriction differ from those caused by accommodation, which excludes the sphincter pupillae muscle as the origin of the signal. By eliminating these muscles as the signal source, we assume to have measured the origin of the accommodation related biopotentials to the ciliary muscle. This consideration raises the question of whether the measured signal is a neuro- or muscular-electrical signal. To answer this question, the ciliary muscle was paralyzed temporarily in five participants. If the measured signal is of muscular origin, it should no longer be possible to record a signal due to paralysis. By applying the cycloplegia Cyclopentolate 1%, the receptors of the ciliary muscle are blocked against the parasympathetically released neurotransmitter acetylcholine. Comparison of the biopotentials with the previous measurements nevertheless shows no difference in 3 out of 5 participants. We also exclude the sympathetic nervous system as the source, as the ciliary muscle is mainly intervened by parasympathetic nerve endings^37^ and the sympathetic part is characterized as relatively slow (30 to 40 s) and with low accommodation changes (≤ 2.0 D)^38^ having no significant involvement in rapid focusing^39^.

Beside probably the most common cycloplegic agent, cyclopentolate 1%^40^ , other commonly used agents such as tropicamide and atropine were also considered to clarify the origin of the signal. The ideal cycloplegic agent and its application are continuously discussed in the literature^40–43^. Atropine is regarded as the gold standard for achieving complete cycloplegia^41,42,44^ especially when administered over a period of 3 days^42^. However, it effects persist for 8–14 days^42^ and the risk of side effects is seven times higher than with cyclopentolate^45^. The latter reaches the maximum effect within 30^42^ to 45^43^ minutes, remains stable for 90 minutes^43^and lasts only for several hours^42^. According to previous studies, the difference in cycloplegic effect between atropine and cyclopentolate is not clinically relevant^46^ or significantly different^42,47^. Tropicamide the second considered cycloplegia, reaches the maximum effect within 30 min, with an effective duration of 75 minutes^43^. However, the effectiveness of tropicamide compared to cyclopentolate is debated. Some studies^40,48^ report a higher residual amount of accommodation with tropicamide than with cyclopentolate, while others describe little to no difference^43,44,49,50^. In order to avoid prolonged cycloplegia and to conservatively ensure a high level of cycloplegia, we decided in favor of cyclopentolate.

This study also has some limitations that should be considered. Firstly, the use of a one-size-fits-all scleral contact lens electrode represents a compromise between stable fit and signal quality due to individual variations in scleral curvature. Some participants reported an increased foreign body sensation when wearing the contact lens electrode, resulting in frequent blinking and movement artifacts, which had a negative impact on signal quality. Also, the empirically determined bandpass blink filter could slightly influence the shape of the recorded signals, although this effect is considered negligible. Secondly, the amplitude measurements between different near targets showed no significant differences, possibly due to lack of accommodation and non-linear changes in refractive power. The standardized measurement procedure with a constant sequence of trigger intervals and the measurement of the refractive change instead of the actual refractive power limits the possibility of assessing training effects and fatigue. It should also be emphasized at this point that our assumption regarding the neurological origin of the signal needs to be further investigated with a larger sample. In our study, the paralyzing cyclopentolate was administered to only 5 participants. In addition, the measurement unit allows data acquisition at 125 samples per second, which may not be sufficient to capture the high-frequency components of the neuroelectric signals, limiting the ability to completely characterize these signals.

## Conclusion

Previous studies have demonstrated the feasibility of recording ciliary muscle electrical potentials during accommodation using various types of electrodes, ranging from invasive needle electrodes inserted into the ciliary muscle to less invasive approaches using contact lens electrodes. Inspired by the findings of these studies, we aimed not only to replicate them using a previously newly developed bipolar scleral lens electrode, but also to quantify and compare the recorded signals with the actual refractive change of the crystalline lens in response to varying accommodative demands. Furthermore, we analyzed the recorded signals for potential confounding sources. Using the exclusion procedure, we postulate that the signal origin is at least to a certain extent of neurological origin. In upcoming studies, we will focus on characterizing accommodation-related signals in presbyopes. In addition, we will test whether these measured signals can be used to control a visual aid with variable refractive power to realize a biomimetic system.

## Methods

### Study participants

**Fig. 6:**
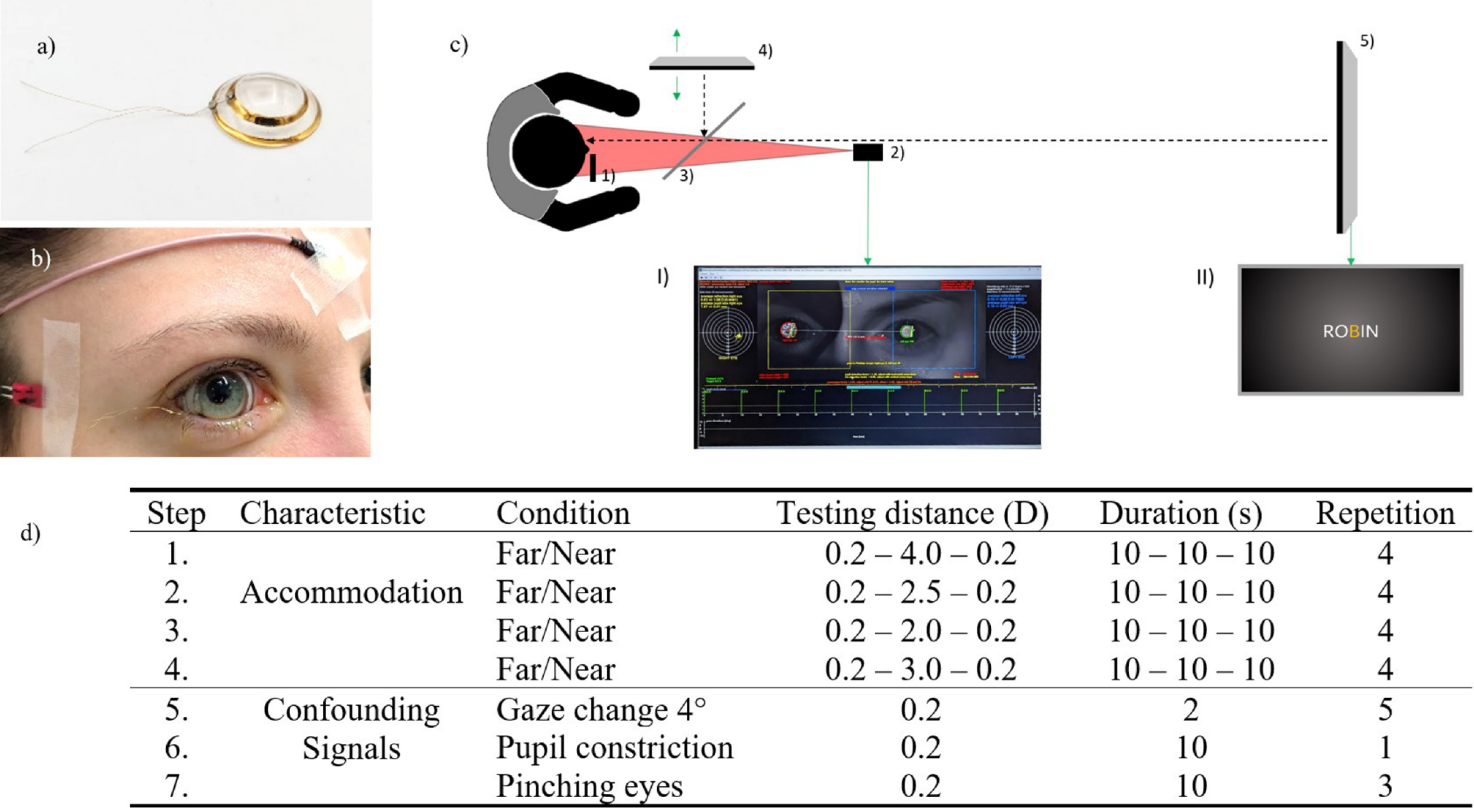
Measurement setup: (**a**) A scleral lens electrode without a connector for the measurement unit (**b**) A participant with a contact lens electrode and a reference electrode on the temple. The gold cables are guided out via the angulus oculi lateralis of the right eye and fixed with a tap. (**c**) Bird’s eye view of the measurement setup. The infrared filter (1) enabling monocular vision but a binocular measurement for the eccentric infrared photorefraction (2). The semi-transparent mirror (3) is light-transmissive when illuminated from the far target. The near target (4) can be adjusted in distance (green arrow), while the far target (5) has a fixed distance of 4.84 m. (**c.a**) Graphical user interface of the custom-made eccentric infrared photorefraction. (**c.b**) Exemplary, a five-digit word presented to the participant. (**d**) Single measurement steps at the testing protocol. Besides examining the accommodation behavior, possible confounding signals were also measured.

12 emmetropic volunteers (5 males, age 24.6 (21–29 years)) were recruited from the student body of the University of Tuebingen after fulfilling all inclusion (age: 18–30 years; spherical equivalent refractive error: < ± 0.5 D; Snellen visual acuity: ≧ 20/20; astigmatism: < 2.0 D; accommodation amplitude: > 4 D) and no exclusion (pregnant, hereditary eye disease, previous or current eye injury, strabismus, amblyopia, pseudophakia) criteria. Power estimation (a = 0.05, power = 0.80, two-tailed matched-pairs t-test) based on the amplitudes recorded during a preliminary study (accommodative effort: 0.5 to 2.0 D)^28^ yielded an effect size of Cohen’s d = 1.3, corresponding to a minimum of 7 participants. To balance robustness and account for potential dropouts while maintaining the feasibility of a pilot study, the sample size was set at 12, also in accordance with Julious (2005)^51^. In participants receiving cycloplegia, intraocular pressure was confirmed to be less than 20.5 mmHg. All participants were shown the contact lens electrode (Fig. 6a) and its dimensions before they received the informed consent form and gave their written consent for voluntary participation. The study was approved by the Ethics Committee of the Medical Faculty of the University of Tuebingen (690/2017BO2) and followed the tenets of the Declaration of Helsinki. Written consent to publish the photo in an open access publication was also obtained from the participant shown in Fig. 6b.

### Preliminary examination

In all participants, objective refraction was measured with an autorefractometer (AR-330 A/AR-360 A, NIDEK CO., LTD., Gamagori, Japan), and uncorrected monocular visual acuity was assessed with a Landolt C chart. Subsequently, the dominant eye was determined by the Dolman hole-in-card test^52,53^. The amplitude of accommodation of the left eye was measured by a motorized push-up method (v = 2 cm/s), focusing on a Duane’s figure as the image target and repeated three times. Finally, a slit-lamp examination of the anterior eye segment and fundus was performed. A Schirmer test II (Schirmer Tear Test - Mark Blue, Optitech Eye Care, Prayagraj, India) was done in the right eye for later analysis by mass spectroscopy^54 ^to determine inflammatory markers before scleral contact lens insertion (as baseline) and after its removal. Participants were asked to close their eyes to reduce discomfort. To ensure a sufficient wetting, the paper strip was collected when reaching more than 15 mm of wetting or else after a five-minute waiting period. Attendees participating in the cyclopentolate measurement additionally underwent intraocular pressure measurements (AT 900, Haag-Streit AG, Koeniz, Switzerland) before and after the actual experiment.

### Experimental setup and stimulus paradigm

The setup, located in a dimly lit room (about 25 lx), has been described before^29^. In brief: It consists of a combined chin and head rest, a high-resolution near-fixation display (2048 × 1536, Adafruit Qualia 9.7” DisplayPort monitor, Adafruit, New York, USA) with adjustable distance (25, 33, 40, and 50 cm), and a far monitor (1360 × 768, NOLK-FD32HB-PNAZ, Richardson Electronics) located at 4.84 m. The axis of the near and the far display are perpendicular to each other and a 45-degree semitransparent mirror allows the stimuli to be superimposed on the visual axis of the fixating eye (Fig. 6c). The non-fixating right eye with the scleral contact lens electrode is covered by an optical long-pass filter (780 nm, RG780, Schott AG, Mainz, Germany). To induce accommodation, five-letter words are randomly drawn from a predefined list and presented at a frequency of 2 Hz in white-on-black Sloan font, with the middle letter highlighted in orange as a fixation aid, alternately for 10 s on the near or the far display at a constant viewing angle. The control of the monitors and the stimulus were implemented in PsychoPy 2022.2.4^55,56^. A high-resolution camera allows to record gaze, pupil diameter, and the refractive change of the crystalline lens during the test using eccentric infrared photorefraction at a sampling rate of 40 Hz^30,57^.

### Participant Preparation

Two drops of local anesthetic (0.4% Novesine^®^, OmniVision GmbH, Puchheim, Germany) were administered to the participant’s right eye. Approximately 2 cm² of the forehead was cleaned with an abrasive paste (Everi Abrasive Conductive Paste, Spes Medica s.r.l., Genoa, Italy) before a gold cup electrode filled with conductive paste (Elefix Z-401CE, Nihon Kohden Corporation, Tokyo, Japan) was placed on the forehead. The bipolar scleral contact lens electrode was filled with a tear substitute (Vidisic, Bausch & Lomb, Laval, Canada) by the ophthalmologist prior to insertion into the right eye to protect the cornea and improve electrical conductivity. During this procedure, the ophthalmologist ensured that the gold wires were guided out of the lateral canthus before firmly taping them onto the participant’s temple. Participants undergoing temporary ciliary muscle paralysis underwent the same procedure except that two drops, as recommended by the manufacturer for diagnostic procedures, of a cycloplegic agent (Cyclopentolate Alcon^®^ 1%, Alcon, Geneva, Switzerland) were instilled 5 minutes apart instead of the topical anesthetic. For maximum cycloplegia, cyclopentolate 1% was administered^58 ^and an instillation time of 20 minutes was given after the second drop. Once the inability to accommodate at near was established by the motorized accommodation ruler, topical anesthetic was administered immediately before the lens electrode insertion.

### Experimental procedure

The experiment included the following measurements, being subsequently performed in each participant. Firstly, to characterize the accommodation signals, an alternating far-near-far stimuli between the far target 0.2 D to different near targets (4.0, 2.5, 2.0, 3.0 D) was shown four times (cf. Figure 6d, Step: 1–4). Subsequently, after the testing distance of 3.0 D, to asses potential confounding signals, horizontal gaze change, pupil constriction and voluntary eye squinting were recorded (cf. Figure 6d, Step: 5–7), before an ophthalmologist removed the contact lens electrode. The experimental procedure ended with the examination of the cornea for abnormalities using the slit lamp and a second Schirmer test II to detect possible inflammatory markers caused by the scleral contact lens. A waiting period of at least three days was specified for the repeat measurement.

### Sample preparation of tear fluid

Punches of Ø 4 mm were taken from the Schirmer strips and transferred into 2.0 mL microcentrifuge tubes. Proteins were extracted as described previously^59^. The protein pellet was resuspended in 30 µL of 50 mM ammonium bicarbonate (ABC), 4 µL of RapiGest SF Surfactant, and 1 µL of 0.1 M dithiothreitol (DTT), and incubated at 60°C for 10 min. After cooling to room temperature, 1 µL of 0.3 M iodoacetamide (IAA) was added, and the mixture was incubated in the dark for 30 min. For enzymatic digestion, 1 µL of 0.5 µg/µL Trypsin/LysC mix was added and incubated overnight at 37°C. The reaction was stopped by adding 3.2 µL of trifluoroacetic acid (TFA) to achieve a final concentration of 5%, followed by centrifugation at 9,000 g for 1 min. The mixture was transferred to a polypropylene insert in a 1.5 mL tube, incubated for 10 min at room temperature, and centrifuged at 16,000 g for 15 min. The clear solution was carefully transferred into new 0.5 mL tubes, avoiding the bottom pellet and upper oily phase. Peptides were cleaned and desalted using StageTips. These Tips were equilibrated with 20 µL of 80/5 solution (80% acetonitrile/5% TFA) and rinsed with 20 µL of 0/5 solution (5% TFA in water). The sample was applied to the StageTip, washed with 20 µL of 0/5 solution, and eluted with 20 µL of 50/5 solution (50% acetonitrile/5% TFA) followed by 20 µL of 80/5 solution. The eluates were pooled and concentrated to 1–5 µL using a SpeedVac concentrator, then stored at -80°C until LC-MS/MS analysis.

### Mass spectrometry

Mass Spectrometry analysis was performed on an Ultimate3000 RSLC system coupled to an Orbitrap Tribrid Fusion mass spectrometer. Tryptic peptides were loaded onto a µPAC Trapping Column (COL -TRPNANO16G1B2, Thermo Fisher Science Inc., Waltham MA, United States). Peptides were eluted and separated on a nano-LC column (µPAC.C18 COLNANOØ5OGIB, Thermo Fisher Science Inc., Waltham MA, United States). The remaining peptides were eluted by a short gradient from 30 to 95% buffer B; the total gradient run was 120 min.

Spectra were acquired in DIA (Data Independent Acquisition) mode using 50 variable-width windows over the mass range 350–1500 m/z. The Orbitrap was used for MS1 (precursors) and MS2 (fragments) detection, with an AGC target for MS1 set to 20 × 104 and a maximum injection time to 100 ms. MS2 scan range was set between 200 and 2000 m/z, with a minimum of 6 points across the peak. Orbitrap resolution for MS2 was set to 30 K, isolation window set to 1.6, AGC target to 50 × 104 and maximum injection time to 54 ms. MS1 and MS2 data were acquired in centroid mode.

To reduce the possibility of carryover and cross-contamination between the samples, one TRAP and two BSA washes were used between samples.

### Data processing

Time series data of pupil diameter, gaze, and lens refractive changes recorded with the eccentric infrared photorefraction were upsampled to 250 Hz to match the sampling frequency of the biopotential amplifier. The biopotential data were band-pass filtered between the empirically determined range of 0.025 and 0.575 Hz with a second-order Butterworth filter to remove blink artifacts and correct biodrifting. Utilizing the average biopotential amplitude from the first 10 s of each measurement, baseline-correction was performed. Thereafter, the biopotential and the eccentric infrared photorefraction data were merged using the trigger signal. A self-developed Python script was created for this step of pre-processing. Two measurements (2.0 D, 4.0 D) of one participant (ID: 02) were excluded due to artifacts with biopotential data exceeding the average value by a factor of twenty.

Data from the far-near-far measurements (Fig. 6d, Step:1–4) were filtered based on pupil diameter and refractive changes, removing values more than two standard deviations from the mean. Additionally, data points where the horizontal or vertical gaze was outside the 95% confidence interval of the mean were also excluded. If the cables were accidentally swapped at the connections, the polarity of the signal was reversed accordingly.

### Filtering and statistics

All biopotential analyzes were done in JMP 16.0.0 (SAS Institute GmbH, Heidelberg, Germany) and 0.05 was defined as the critical alpha value for statistical assessments.

The mean of each 10 s sequence from the data of the far-near-far measurements (Fig. 6d, Step: 1–4) for biopotential and refractive change was calculated. Effects of these dependent variables were analyzed by linear mixed-effects models using restricted maximum likelihood (REML). As independent variables, accommodation demand (4.0 D, 2.5 D, 2.0 D, and 3.0 D), session as well as their interaction were set. To account for inter individual variability and repeated measurements participant was declared as a random effect. Knowing the robustness of linear mixed-effects models towards deviations from normality^60,61^ the model’s conditional residuals were verified (pq-plot, skewness, kurtosis) for at most moderate deviations from normal distribution. Homogeneity of the variances was ensured using the Brown–Forsythe test and reported in case of violations. Post hoc comparisons of the least squares mean were conducted using two-tailed Tukey HSD.

Due to the characteristic S-shape consisting of the three sections gradual onset, rapid change and stabilization, the logistic sigmoid function $\:{sig}_{\left(t\right)}$was used for mathematical description of the accommodation-related signals over time (*t*). This involved initial normalization of the filtered signals (0 to 1) for better visualization. In order to obtain a complete picture of the accommodation and disaccommodation process, the time interval was set to 5 s before and after the respective stimulus.

1 $$\:{\varvec{s}\varvec{i}\varvec{g}}_{\left(\varvec{t}\right)}=\varvec{c}+\frac{\varvec{d}-\varvec{c}}{1+{\varvec{e}}^{-\varvec{a}\left(\varvec{t}-\varvec{b}\right)}}$$.

To describe the range of speed in the confounding signals measurement, the delta of the y-value (in mV) was determined at time point 0 and 2 s and divided by the elapsed time of 2 s.

Before determining the repeatability between the measurements with and without cyclopentolate utilizing a Bland-Altman plot and a two tailed t-test the sequence means were checked for normal distribution (pq-plot, skewness, kurtosis).

The DIA MS RAW data were analyzed using DIA-NN 1.8.1 (PMID: 31768060) in library-free mode against the human database (UniProt release November 2023, 20405 proteins). First, a precursor ion library was generated using FASTA digest for library-free search in combination with deep learning-based spectra prediction. An experimental library generated from the DIA-NN search was used for cross-run normalization and Mass accuracy correction. Only high-accuracy spectra with a minimum precursor FDR of 0.01, and only tryptic peptides (2 missed Tryptic cleavages) were used for protein quantification. The match between runs option was activated and no shared spectra were used for protein identification. Data was analyzed and processed using the Perseus platform^62^.

## Electronic supplementary material

Below is the link to the electronic supplementary material.

Supplementary Material 1

Supplementary Material 2

Supplementary Material 3

## Data Availability

The datasets generated during the current study are available from the corresponding author on reasonable request.
