# Supplementary material for: Non-invasive measuring of biopotentials of the ciliary muscle during accommodation in emmetropes
Source: Sci Rep. 2025 Jun 3;15:19389. doi: 10.1038/s41598-025-04165-3 (PMC12134129; doi:10.1038/s41598-025-04165-3)
Supplement: Supplementary file 1 — Supplementary Material 1 [file 41598_2025_4165_MOESM1_ESM.docx]

| 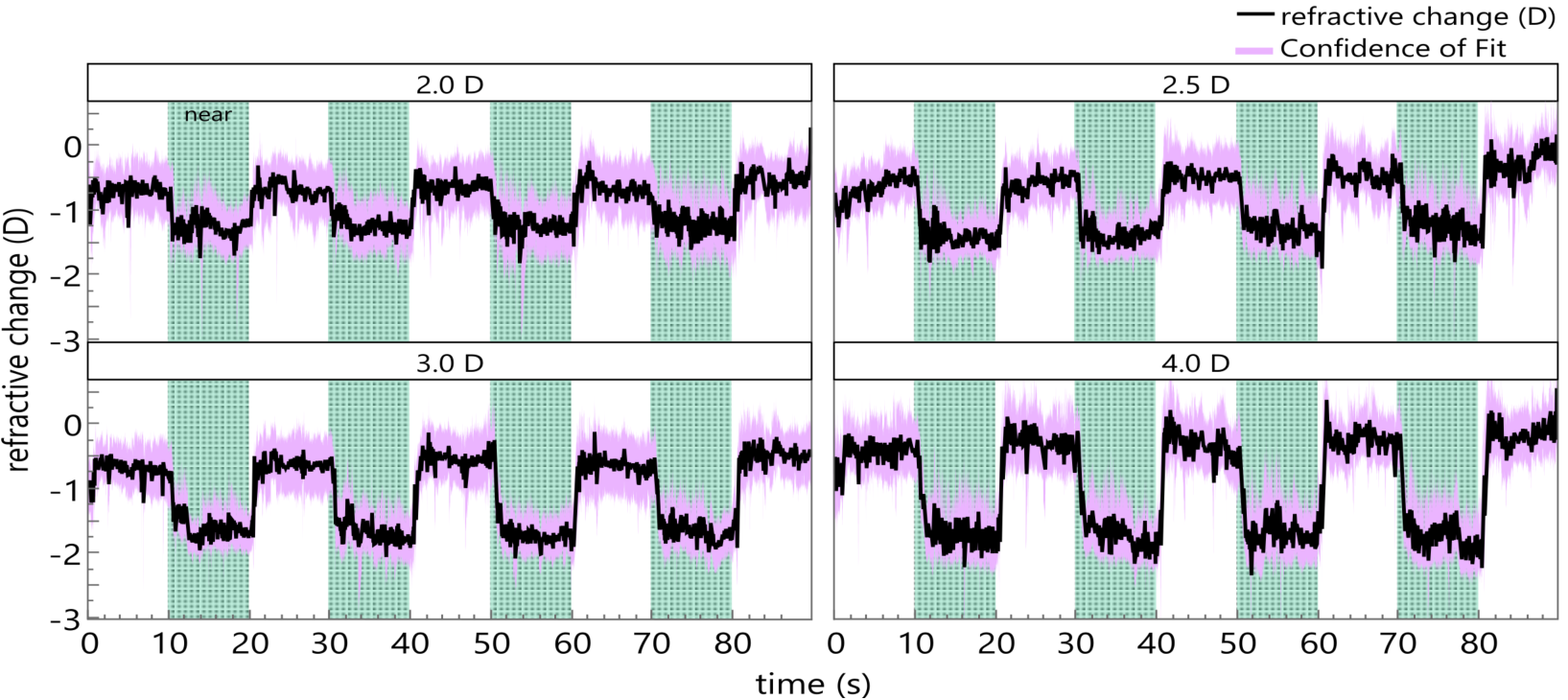  a) |
| --- |
| 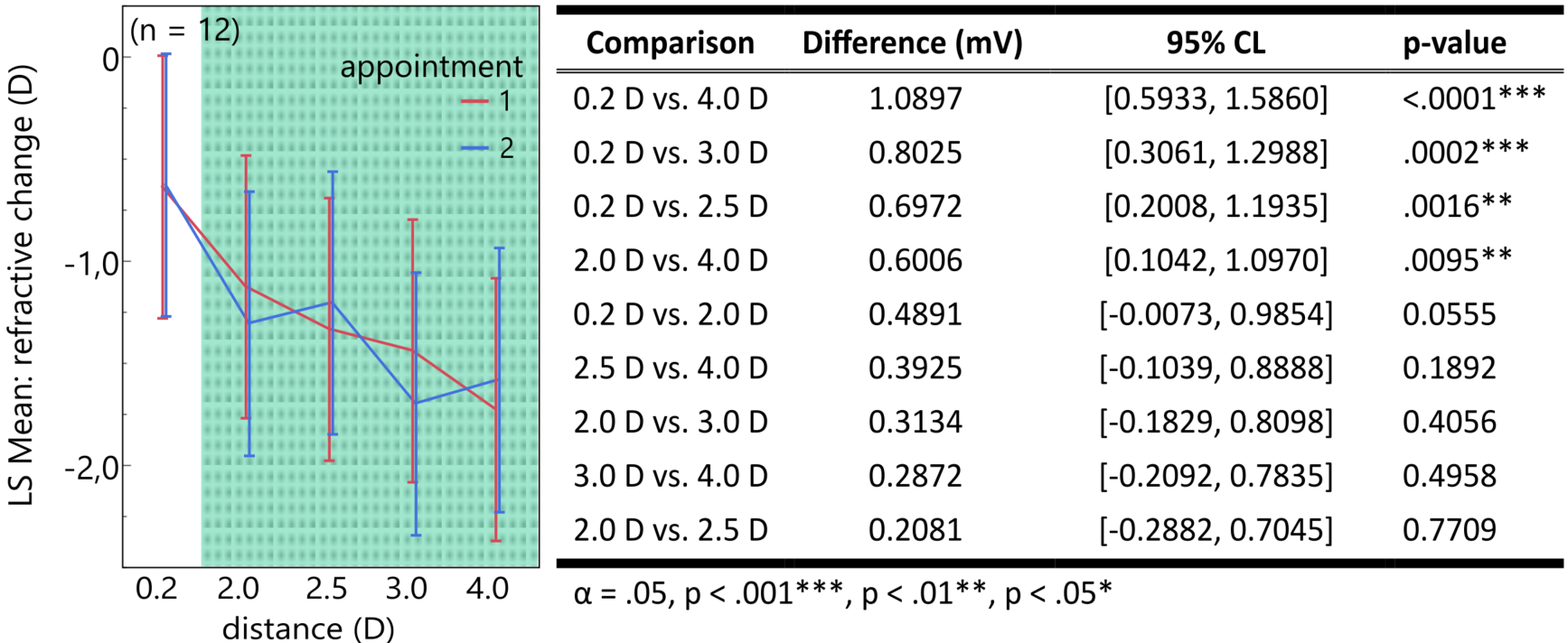  b) |
| Supplementary Figure S1: a) Moving average of the refractive change (width:10) of the two measurement appointments for all participants^x^ with the purple shaded area as the 95%-confidence of fit. The greenish marked background represents the 10 seconds near sequences at different near distances (2.0, 2.5, 3.0, 4.0D). b) (left) The LS mean plot of the electrical potential amplitude over the different accommodative demands for the first and second appointment. (right) Results of the post hoc Tukey-HSD test where the mean amplitude between different accommodative demands are compared. ^x^Measurements (ID:02 2.0D & ID:02 4.0D) were excluded. |

**Supplementary Information**

| 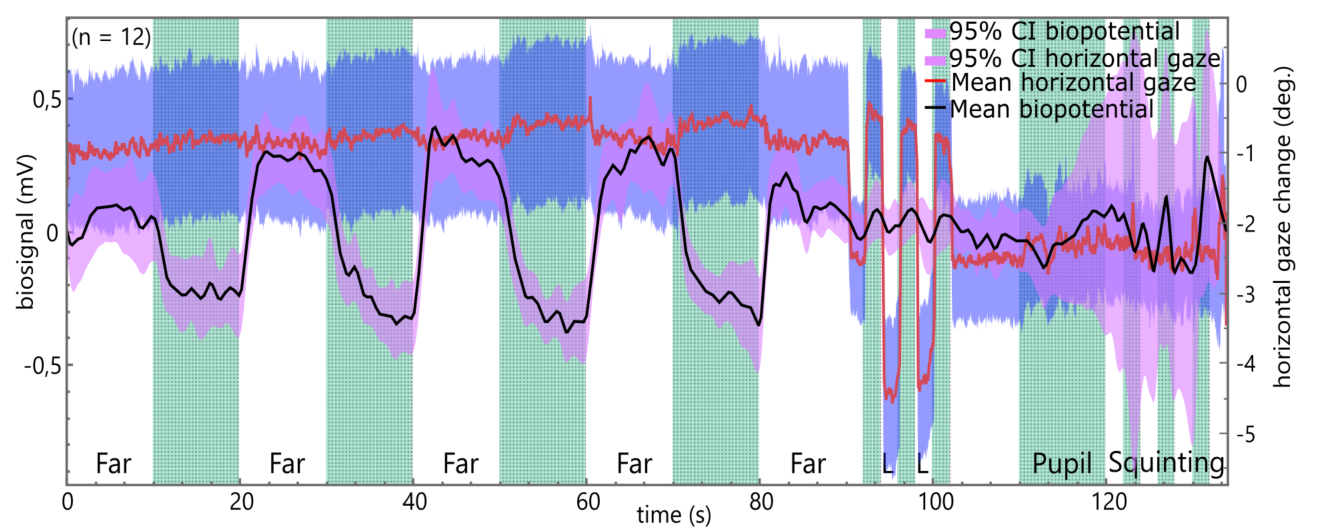 |
| --- |
| Supplementary Figure S2: The mean biopotential (black line, 95% confidence in purple) of the two measurements is represented by the left y-axis, whereas the right y-axis shows the respective horizontal gaze change (red line, 95% confidence in blue) over the entire confounding signal measurement. During the alternating change between far and near the gaze is relatively stable, due to the calibrated aligned axis of both monitors. By triggering eye movement, the horizontal gaze changes about 4°, whereas the biopotentials alters far less than during the accommodation-related changes. |
