# Supplementary figures and images for: Non-invasive measuring of biopotentials of the ciliary muscle during accommodation in emmetropes
Source: Sci Rep. 2025 Jun 3;15:19389. doi: 10.1038/s41598-025-04165-3 (PMC12134129; doi:10.1038/s41598-025-04165-3)

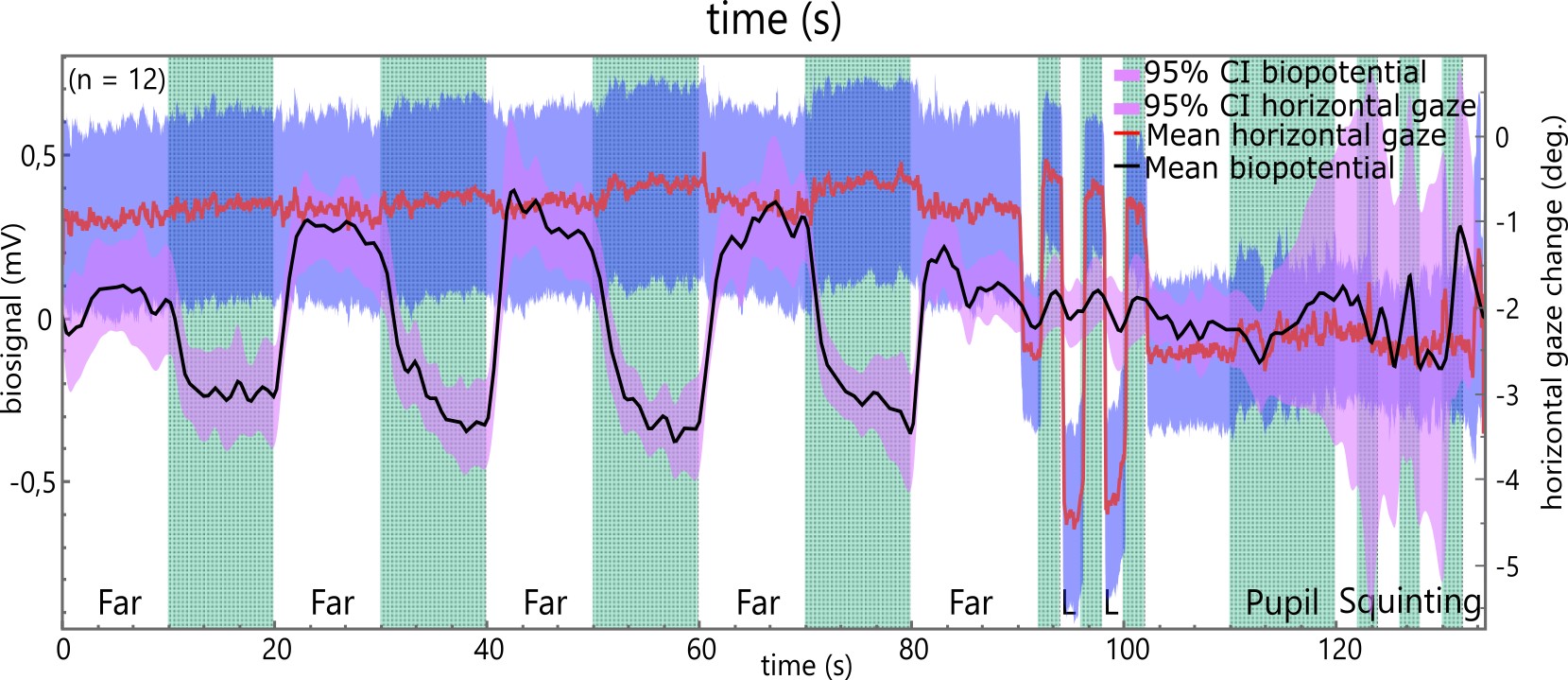

Supplement: Supplementary file 2 — Supplementary Material 2 [file 41598_2025_4165_MOESM2_ESM.jpg]

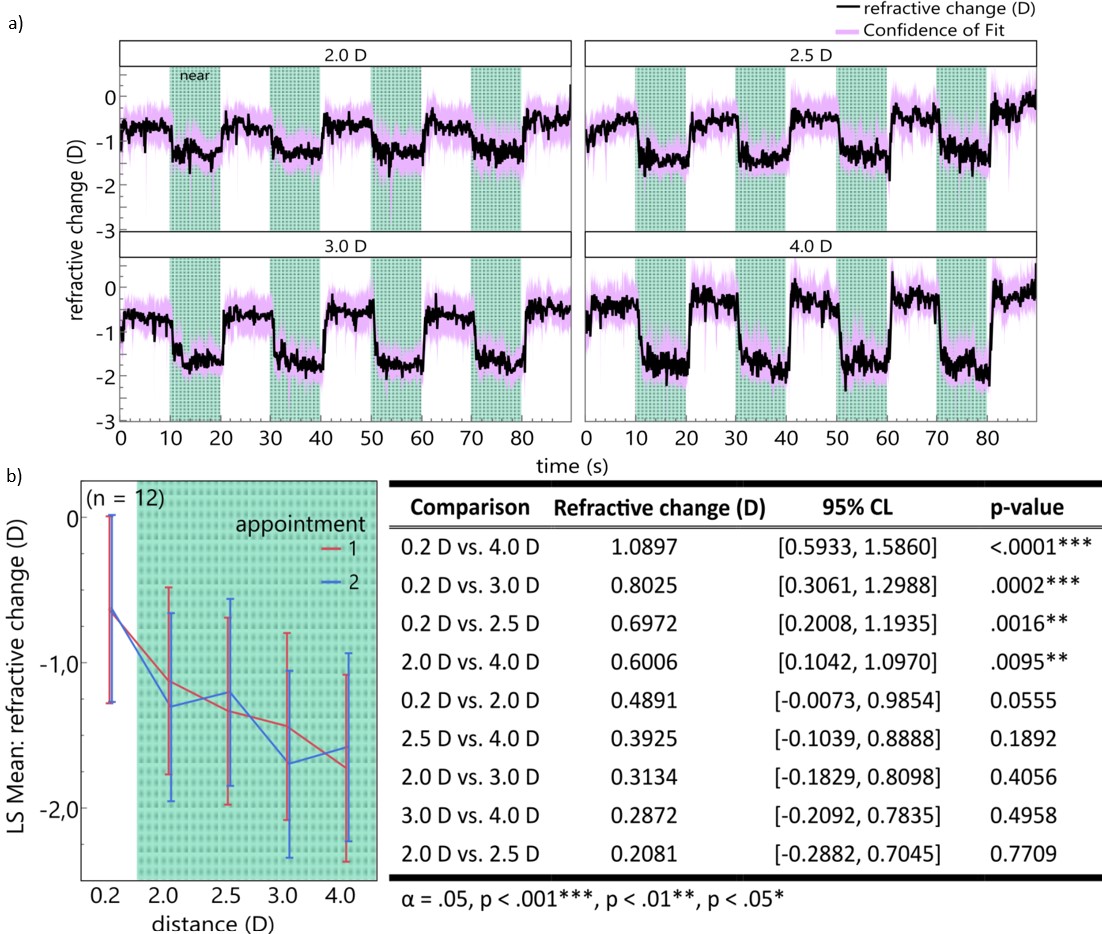

Supplement: Supplementary file 3 — Supplementary Material 3 [file 41598_2025_4165_MOESM3_ESM.jpg]
